# Cathepsin-S degraded decorin are elevated in fibrotic lung disorders – development and biological validation of a new serum biomarker

**DOI:** 10.1186/s12890-017-0455-x

**Published:** 2017-08-09

**Authors:** S.N. Kehlet, C.L. Bager, N. Willumsen, B. Dasgupta, C. Brodmerkel, M. Curran, S. Brix, D.J. Leeming, M. A. Karsdal

**Affiliations:** 1 Nordic Bioscience A/S, Herlev, Denmark; 2 Proscion A/S, Herlev, Denmark; 3 Janssen Pharmaceutical Companies of J & J, LLC, Springhouse, PA USA; 4 Department of Biotechnology and Biomedicine, Technical University of Denmark, Kongens Lyngby, Denmark

**Keywords:** Decorin, Cathepsin-S, Extracellular matrix, Cancer, Idiopathic pulmonary fibrosis, Serum biomarker

## Abstract

**Background:**

Decorin is one of the most abundant proteoglycans of the extracellular matrix and is mainly secreted and deposited in the interstitial matrix by fibroblasts where it plays an important role in collagen turnover and tissue homeostasis. Degradation of decorin might disturb normal tissue homeostasis contributing to extracellular matrix remodeling diseases. Here, we present the development and validation of a competitive enzyme-linked immunosorbent assay (ELISA) quantifying a specific fragment of degraded decorin, which has potential as a novel non-invasive serum biomarker for fibrotic lung disorders.

**Methods:**

A fragment of decorin cleaved in vitro using human articular cartilage was identified by mass-spectrometry (MS/MS). Monoclonal antibodies were raised against the neo-epitope of the cleaved decorin fragment and a competitive ELISA assay (DCN-CS) was developed. The assay was evaluated by determining the inter- and intra-assay precision, dilution recovery, accuracy, analyte stability and interference. Serum levels were assessed in lung cancer patients, patients with idiopathic pulmonary fibrosis (IPF), patients with chronic obstructive pulmonary disease (COPD) and healthy controls.

**Results:**

The DCN-CS ELISA was technically robust and was specific for decorin cleaved by cathepsin-S. DCN-CS was elevated in lung cancer patients (*p* < 0.0001) and IPF patients (*p* < 0.001) when compared to healthy controls. The diagnostic power for differentiating lung cancer patients and IPF patients from healthy controls was 0.96 and 0.77, respectively.

**Conclusion:**

Cathepsin-S degraded decorin could be quantified in serum using the DCN-CS competitive ELISA. The clinical data indicated that degradation of decorin by cathepsin-S is an important part of the pathology of lung cancer and IPF.

## Background

Idiopathic pulmonary fibrosis (IPF), chronic obstructive pulmonary disease (COPD) and lung cancer are lung pathologies which are characterized by excessive accumulation of extracellular matrix (ECM) leading to loss of tissue homeostasis and progressive disease phenotype [1–3]. Biomarkers which reflect these processes may therefore play an important role in identifying patients with rapid disease progression. Decorin is a member of the small leucine-rich proteoglycan (SLRP) family and is one of the most abundant proteoglycans of the interstitial matrix. The protein is mainly secreted and deposited by fibroblasts. It consists of a single covalently attached N-terminal glycosaminoglycan (GAG) chain, composed of either dermatan or chondroitin sulfate, and 12 leucine-rich tandem repeats representing the protein core [4–6].

Due to its diverse ECM protein binding partners and its regulation of cell growth and cell differentiation, decorin has been named as “the guardian from the matrix” [7] recognizing the significance of decorin in tissue homeostasis. The main ECM binding partners are fibrillar collagens (type I, II, III and VI) and decorin has shown to play a role in the regulation of fibrillogenesis and stabilization of fibrils, and may act as a central player in collagen assembly/turnover and consequently tissue homeostasis [8, 9]. Supporting this, decorin knock-out in mice results in abnormal collagen fibril formation and enhanced collagen degradation [10].

In addition to playing a role in collagen fibril formation in the interstitial ECM, decorin sequesters multiple growth factors, such as TGF-beta and directly antagonizes several members of the receptor tyrosine kinase family, including the epidermal growth factor receptor (EGFR) and insulin-like growth factor receptor I (IGF-IR) [7, 11–14]. As a consequence, decorin regulates survival, migratory, proliferative and angiogenic signaling pathways.

Decorin’s ability to modulate various signal transduction pathways has given it a valid reputation within cancer and several studies have revealed decorin as a tumor repressor which counteracts tumorigenic and angiogenic growth [15]. Furthermore, reduced decorin within the tumor stroma is a poor prognostic factor of invasive breast-, lung- and soft tissue cancers as well as in myeloma [5, 16–18].

Decorin appears to have a protective role in cancer and has also been shown to have anti-fibrotic properties. Fibrosis is characterized by an increased and disorganized deposition of ECM proteins resulting in loss of tissue and organ function. One of the key pro-fibrotic mediators is TGF-beta, a chemotactic factor for fibroblasts enhancing the synthesis of ECM proteins. As decorin is an inhibitor of TGF-beta, numerous studies have investigated the decorin’s potential to block the fibrotic response and decorin has shown to reduce tissue fibrosis in kidney and lung in multiple disease models [19–21].

Increased ECM remodeling and protease-mediated degradation of ECM proteins is a well-documented and significant component of cancer pathology and lung fibrosis [1–3, 22]. We hypothesize that degradation of decorin may have biomarker potential in these pathologies as degradation of decorin might inactivate and disrupt its anti-tumor and anti-fibrotic capabilities.

A decorin fragment was previously identified in human knee articular cartilage using mass spectrometry (MS/MS) [23]. The aim of this work was to develop a competitive enzyme-linked immunosorbent assay (ELISA) targeting the specific degraded fragment of decorin, identify the protease generating this fragment, and to investigate the biomarker potential of this fragment in serum from patients with various lung pathologies.

## Methods

### Selection of peptides

The following cleavage site (↓) on decorin was previously identified in human knee articular cartilage using mass spectrometry and published by Zhen et al. [23]: _72_LDK↓VPKDLPPDTT_84_ located in the first leucine rich repeat of the protein.

In order to generate an antibody specific for the N-terminal of the cleavage fragment, a sequence of 10 amino acids adjacent to the site was chosen as the target: ↓_75_VPKDLPPDTT_84_. This amino acid sequence was used to design the standard peptide. The sequence was blasted for homology to other human secreted extracellular matrix proteins using NPS@: Network Protein Sequence Analysis with the UniprotKB/Swiss-prot database [24].

Synthetic peptides used for monoclonal antibody production and validation of the ELISA assay were purchased from Chinese Peptide Company (Hangzhou, China) and Genscript (Piscataway, NJ, USA) and shown in Table 2. A biotinylated peptide (VPKDLPPDTT-biotin) was included as a coating peptide on streptavidin-coated ELISA plates. The specificity of the antibody was tested by including an elongated standard peptide with an additional amino acid added to the N-terminal of the peptide sequence (KVPKDLPPDTT), as well as a non-sense standard peptide (DSSAPKAAQA) and a non-sense biotinylated coating peptide (biotin-DSSAPKAAQA) in the assay validation. The immunogenic peptide (VPKDLPPDTT-KLH) was generated by covalently cross-linking the standard peptide to Keyhole Limpet Hemocyanin (KLH) carrier protein using Succinimidyl 4-(N-maleimidomethyl)cyclohexane-1-carboxylate, SMCC (Thermo Scientific, Waltham, MA, USA, cat.no. 22336).

### Monoclonal antibody production

Four to six week old Balb/C mice were immunized by subcutaneous injection of 200 μL emulsified antigen containing 50 μg immunogenic peptide (VPKDLPPDTT-KLH) mixed with Freund’s incomplete adjuvant (Sigma-Aldrich, St. Louis, MO, USA). Consecutive immunizations were performed at 2-week intervals until stable sera titer levels were reached. The mouse with the highest titer rested for four weeks and was then boosted with 50 μg immunogenic peptide in 100 μL 0.9% NaCl solution intravenously. Hybridoma cells were produced by fusing spleen cells with SP2/0 myeloma cells as previously described [25]. The resultant hybridoma cells were then cultured in 96-well microtiter plates and standard limited dilution was used to secure monoclonal growth. The supernatants were screened for reactivity using the biotinylated peptide (VPKDLPPDTT-biotin) as coating agent in the competitive immunoassays.

### Clone characterization

The reactivity of the monoclonal antibodies was evaluated by displacement using human serum samples and the standard peptide (VPKDLPPDTT) in a preliminary ELISA using 10 ng/mL biotinylated coating peptide on streptavidin-coated microtiter plates (Roche, Basel, Switzerland, cat. #11940279) and the supernatant from the antibody producing monoclonal hybridoma cells. The clone with the best reactivity towards the standard peptide was purified using protein-G-columns according to the manufacturer’s instructions (GE Healthcare Life Sciences, Little Chalfont, UK, cat. #17–0404-01).

### Cleavage of decorin in vitro

Reconstituted human recombinant decorin (ACRO Biosystems, Newark, DE, USA, cat. # DE1-HS223) was diluted to a final concentration of 100 μg/mL in cathepsin buffer (100 mM sodium phosphate, 2 mM DTT, 0.01% Brij-35, pH 7.4), MMP-buffer (50 mM Tris-HCL, 200 mM NaCl, 10 mM CaCl_2_, 100 μM ZnAc, pH 7.5) or ADAMTS-5 buffer (50 mM Tris, 100 mM NaCl, 5 mM CaCl_2_, 0.05% Brij-35, pH 7.5). The solutions incubated at 37 °C for 1 h, 24 h and 72 h with or without the addition of the following proteases: Cathepsin-S (Merckmillipore, cat. # 219343), cathepsin-L (Merckmillipore, cat. # 219402), APMA activated MMP-2 (Biocol, cat. # II.5), MMP-9 (Giotto, cat. # G04MP09C) and ADAMTS-5 (R&D systems, cat. # 2198-AD). Cathepsins and MMPs were added to a final concentration of 2 μg/mL and ADAMTS-5 to a final concentration of 10 μg/mL. A positive control protein with known cleavage by the above proteases was included. The reaction was stopped by adding E-64 (final concentration of 1 uM) to cathepsins solutions and EDTA to the MMP solutions (final concentration of 1 uM) and ADAMTS-5 solutions (final concentration of 5 uM). Cathepsin-, MMP- and ADAMTS-5 buffer with relevant proteases were included as controls. Samples were stored at −80 °C until analysis. The cleavage of decorin was confirmed by silverstaining according to the manufacturer’s instructions (SilverXpress®, Invitrogen, cat. #LC6100) and coomassie blue (data not shown).

### DCN-CS (decorin degraded by cathepsin-S) ELISA protocol

Optimal incubation -buffer, −time and -temperature, as well as the optimal concentrations of antibody and coating peptide were determined and the finalized DCN-CS competitive ELISA protocol was as follows:

A 96-well streptavidin-coated microtiter plate was coated with 2.5 ng/mL biotinylated coating peptide dissolved in assay buffer (50 mM Tris-BTB, 4 g/L NaCl, pH 8.0) and incubated for 30 min. at 20 °C in darkness shaking (300 rpm). Twenty μL standard peptide or pre-diluted serum (1:4) were added to appropriate wells, followed by the addition of 100 μL monoclonal antibody dissolved in assay buffer to a concentration of 30 ng/mL to each well and incubated 20 h at 5 °C in darkness shaking (300 rpm). One hundred μL of goat anti-mouse POD-conjugated IgG antibody (Thermo Scientific, Waltham, MA, USA, cat. #31437) diluted 1:6000 in assay buffer to obtain a final concentration of 130 ng/mL was added to each well and incubated 1 h at 20 °C in darkness shaking. All incubation steps were followed by five washes in washing buffer (20 mM Tris, 50 mM NaCl, pH 7.2). Finally, 100 μL tetramethylbenzidine (TMB) (cat. 438OH, Kem-En-Tec Diagnostics, Denmark) was added to each well and the plate was incubated for 15 min at 20 °C in darkness shaking. The enzymatic reaction was stopped by adding 0.18 M H2SO4 and absorbance was measured at 450 nm with 650 nm as reference. A calibration curve was plotted using a 4-parameter logistic curve fit. Data were analyzed using the SoftMax Pro v.6.3 software.

### Technical evaluation of the DCN-CS ELISA

To evaluate the technical performance of the DCN-CS ELISA, the following validation tests were carried out: Inter- and intra-assay variation, linearity, lower limit of detection, upper limit of detection, lower limit of quantification, analyte stability (freeze/thaw and storage) and interference.

The inter- and intra-assay variation was determined by ten independent runs on different days using ten quality control samples covering the detection range, with each run consisting of double-determinations of the samples. The ten quality control samples consisted of: two human serum samples, one sheep serum sample, one fetal calve serum sample, four human serum samples spiked with standard peptide and two samples with standard peptide in buffer. Intra-assay variation was calculated as the mean coefficient of variance (CV%) within plates and the inter-assay variation was calculated as the mean CV% between the ten individual runs. To assess linearity of the assay, two-fold dilutions of human serum samples were performed and dilution linearity was calculated as a percentage of recovery of the un-diluted sample. The lower limit of detection (LLOD) was determined from 21 measurements using assay buffer as sample and was calculated as the mean + three standard deviations. The upper limit of detection (ULOD) was determined from ten independent runs of the highest standard peptide concentration and was calculated as the mean back-calibration calculation + three standard deviations. The lower limit of quantification (LLOQ) was determined from three independent runs of a serum sample diluted stepwise and determined as the highest DCN-CS level quantifiable in serum with a coefficient of variation below 30%. Analyte stability was first determined by the effect of repeated freeze/thaw of serum samples by measuring the DCN-CS level in three human serum samples in four freeze/thaw cycles. The freeze/thaw recovery was calculated with the first cycle as reference. Second, analyte stability in relation to storage was determined by a 24 h study performed at 4 °C or 20 °C. The DCN-CS level in three human serum samples was measured after 0 h, 2 h, 4 h and 24 h of storage and recovery was calculated with samples stored at −20 °C as reference. Interference was determined by adding a low/high content of hemoglobin (0.155/0.310 mM), lipemia/lipids (4.83/10.98 mM) and biotin (30/90 ng/mL) to a serum sample of known concentration. Recovery percentage was calculated with the normal serum sample as reference.

### Clinical validation of DCN-CS

Patient serum samples consisted of three different cohorts. Cohort 1 was obtained from the commercial vendor ProteoGenex (Culver City, CA, USA) and included patients with non-small cell lung cancer (NSCLC), IPF, COPD and colonoscopy-negative controls with no symptomatic or chronic disease. A panel of healthy donors acquired from the commercial vendor Valley Biomedical (Winchester, VA, USA) were included as controls (Table 1).

**Table 1:** Clinical sample overview and patients demographics

Cohort	Samples	No. of subjects	Mean age (range)	Gender, % females	Tumor stage I	Tumor stage II	Tumor stage III	Tumor stage IV
1	NSCLC patients	8	61 (47–77)	12.8	1	2	3	2
1	IPF patients	8	74 (55–82)	62.5	-	-	-	-
1	COPD patients	8	75 (69–82)	50.0	-	-	-	-
1	Colonoscopy-negative controls	8	55 (44–65)	75.0	-	-	-	-
1	Healthy controls	20	34 (20–51)	10.0	-	-	-	-
2	NSCLC patients^a^	12	60 (47–80)	25.0	5	2	4	-
2	SCLC patients	8	61 (54–82)	25.0	3	1	4	-
2	Healthy controls	43	71 (60–82)	100.0	-	-	-	-
3	IPF patients	116	65 (43–80)	21.5	-	-	-	-
3	Healthy controls	38	34 (20–58)	10.5	-	-	-	-

^a^No tumor stage information of one patient

Cohort 2 consisted of lung cancer patients acquired from the commercial vendor Asterand (Detroit, MI, USA) and healthy control serum samples obtained from a Danish study population.

Cohort 3 was a combination of serum samples from patients diagnosed with IPF (baseline samples, CTgov reg. NCT00786201) and healthy control serum samples acquired from the commercial vendor Valley Biomedical (Winchester, VA, USA) (Table 1).

### Statistical analysis

The level of DCN-CS in serum samples was compared using one-way ANOVA adjusted for Tukey’s multiple comparisons test (parametric data), Kruskal-Wallis adjusted for Dunn’s multiple comparisons test (non-parametric data) or unpaired, two-tailed Mann-Whitney test. D’Agostino-Pearson omnibus test was used to assess the normality of the data. The diagnostic power was investigated by the area under the receiver operating characteristics (AUROC). Sensitivity and specificity were determined for cut-off values based on the ROC curves. The cut-off values should be regarded as a preliminary estimated cut-off point applied to achieve the reported maximized sensitivity and specificity.

Unless otherwise stated, data are shown as Tukey box plots, where the boxes represent the 25th, 50th and 75th percentiles. The whiskers represent the lowest and highest value, except outliers, which are higher than 1.5 times the 75th percentile or lower than 1.5 times the 25th percentile. *P*-values <0.05 were considered significant. Statistical analyses were performed using GraphPad Prism version 6 (GraphPad Software, Inc., CA, USA) and MedCalc Statistical Software version 12 (MedCalc Software, Ostend, Belgium). Graphs were designed using GraphPad Prism version 6 (GraphPad Software, Inc., CA, USA).

## Results

### Specificity of the DCN-CS ELISA assay

The target sequence, _75_VPKDLPPDTT_84_, was blasted for homology to other human secreted extracellular matrix proteins using NPS@: Network Protein Sequence Analysis with the UniprotKB/Swiss-prot database. The target sequence was found to be unique to human decorin when compared to other secreted ECM proteins. Allowing one amino acid mismatch, two secreted extracellular matrix proteins, Wnt-11 and Podocan, were identified with mismatches at the 6th and 4th position, respectively (Table 2). There was no reactivity against the sequence of Wnt-11, whereas some reactivity was observed against Podocan (data not shown). However, the affinity of the antibody was approximately 10 times higher for decorin than the podocan peptide. At the same time, it is unknown whether podocan will be cleaved in vivo between the exact two amino acids creating this peptide fragment. Furthermore, decorin has been shown to be the most abundant proteoglycan in human adult skin [6] decreasing the likelihood of reactivity towards podocan in biological samples.

**Table 2:** Synthetic peptides used for development and validation of the DCN-CS ELISA assay

Peptide name	Amino acid sequence
Selection/standard peptide	VPKDLPPDTT
Immunogenic peptide	VPKDLPPDTT-KLH
Biotinylated coating peptide	VPKDLPPDTT-biotin
Elongated peptide	KVPKDLPPDTT
Non-sense selection peptide	DSSAPKAAQA
Non-sense coating peptide	biotin-DSSAPKAAQA
Wnt-11 peptide	VPKDLDIRPV
Podocan peptide	VPKHLPPALY

**Fig. 1:**
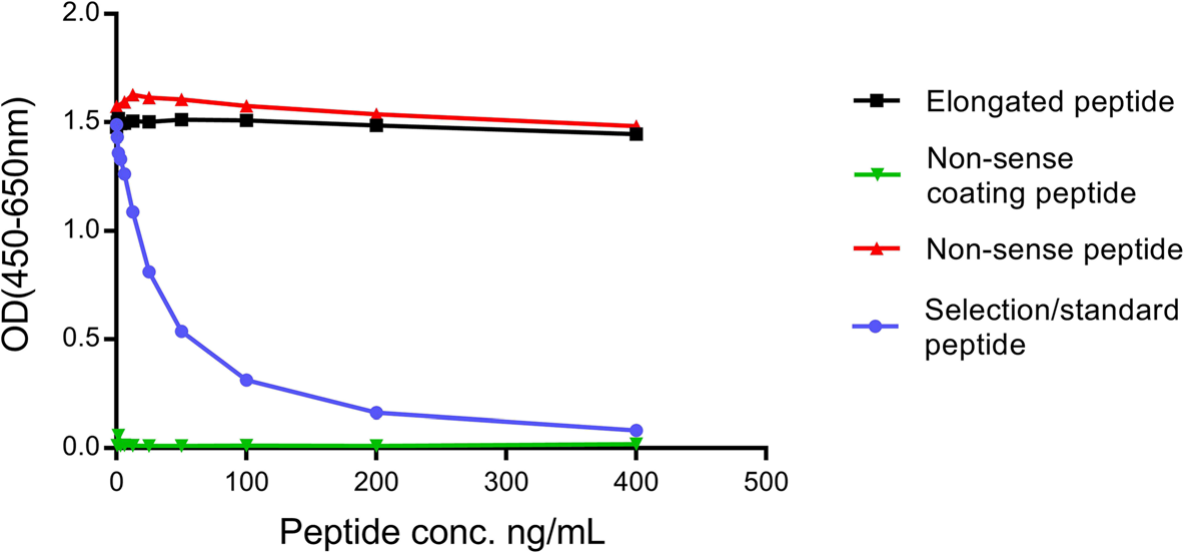
Specificity of the DCN-CS monoclonal antibody. Monoclonal antibody reactivity towards the standard peptide (VPKDLPPDTT), the elongated peptide (KVPKDLPPDTT), a non-sense peptide (DSSAPKAAQA) and a non-sense coating peptide (biotin-DSSAPKAAQA) was tested for in the competitive DCN-CS ELISA assay. Signals are shown as optical density (OD) at 450 nm (subtracted the background at 650 nm) as a function of peptide concentration

The specificity of the competitive DCN-CS ELISA was evaluated by analyzing the reactivity towards the standard peptide, a non-sense peptide, an elongated peptide and using a non-sense biotinylated coating peptide. All peptide sequences are shown in Table 2 and results are shown in Fig. 1. The antibody only reacted with the standard peptide and the standard peptide clearly inhibited the signal in a dose-dependent manner compared to the other peptides. No detectable signal was observed when using the non-sense biotinylated coating peptide. These data suggest that the selected antibody exhibits high epitope specificity.

### Degradation by Cathepsin-S

**Fig. 2:**
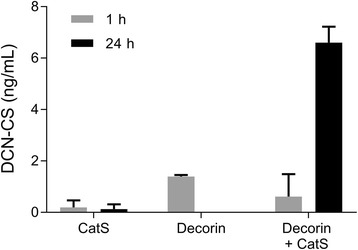
Cleavage of decorin by Cathepsin-S. Degraded decorin levels were measured after 1 h and 24 h incubation of human recombinant decorin with Cathepsin-S. Data were normalized by subtracting the background measured in buffer alone. The experiment was repeated twice and data are shown as the mean of the two replicates with standard deviation

The ability of different proteases to generate the specific decorin fragment was investigated by incubating recombinant human decorin with cathepsin-S (Cat-S), Cathepsin-L (Cat-L), MMP-2, MMP-9 and ADAMTS-5. As shown in Fig. 2, Cat-S was able to generate decorin fragments in a time-dependent manner. Almost 6-fold higher decorin fragments were detected after incubating recombinant decorin with Cat-S for 24 h. No cleavage was observed with Cat-L, MMP-9, MMP-2 and ADAMTS-5 up to 72 h of incubation (data not shown).

Together, these results show that Cat-S can generate the target peptide recognized by the antibody.

### Technical evaluation of the DCN-CS ELISA assay

A series of technical validations were performed to further evaluate the DCN-CS ELISA. The different validation steps and DCN-CS performance are shown in Table 3. The measuring range (LLOD to ULOD) of the assay was determined to 1.2–345.3 ng/mL and the lower limit of quantification (LLOQ) was 5.3 ng/mL. The intra- and inter-assay variation was 3 and 13%, respectively. The acceptance criterion was below 10% for the intra-assay variation and below 15% for the inter-assay variation and therefore acceptable. Human serum needed to be diluted 1:4 to obtain linearity and mean dilution recovery for pre-diluted human serum was 100%. The analyte recovery in serum was 94% after 4 freeze/thaw cycles and after storage at 4 °C for 24 h the recovery was 87%. The acceptance criterion was a recovery within 100% ± 20%. Analyte stability was also tested at 20 °C for 2, 4 and 24 h. The recovery after 2 and 4 h was 93% and 78%, respectively. However after 24 h the analyte could not be recovered within the acceptance range (53% recovery). These data indicate that the analyte in serum is stable at 4 °C and serum samples to be analyzed for DCN-CS should not be stored above this temperature for more than four hours. No interference was detected from either low or high contents of biotin, lipids or hemoglobin with recoveries ranging from 86 to 107%. The acceptance criterion was a recovery within 100% ± 20%.

**Table 3:** Technical validation data of the DCN-CS ELISA assay

Tecnical validation step	DCN-CS performance
Detection range (LLOD-ULOD)	1.2–345.3 ng/mL
Lower limit of quantification (LLOQ)	5.3 ng/mL
Intra-assay variation	3%
Inter-assay variation	13%
Dilution of serum samples	1:4
Dilution recovery (1:4 pre-dilution)^a^	100% (82–113%)
Freeze/thaw recovery (4 cycles)^a^	94% (90–97%)
Analyte stability up to 24 h, 4 °C^a^	87% (86–90%)
Interference Lipids, low/high	107%/86%
Interference Biotin, low/high	100%/100%
Interference Hemoglobin, low/high	100%/100%

^a^Percentages are reported as mean with range shown in brackets

### Clinical evaluation – DCN-CS as a biomarker for fibrotic lung disorders

**Fig. 3:**
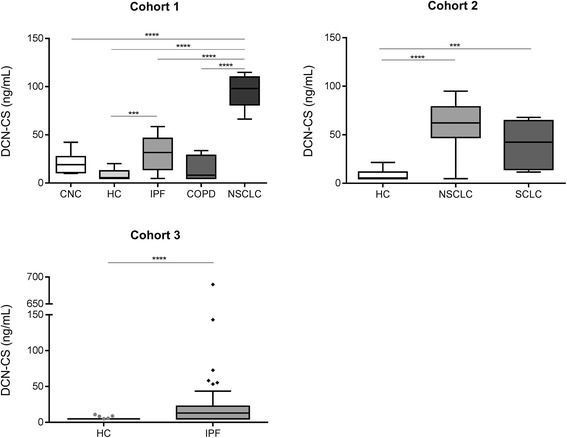
Serum DCN-CS levels in patients with fibrotic lung disorders. Serum DCN-CS was assessed in three independent cohorts: Cohort 1 included patients with NSCLC (*n* = 8), IPF (*n* = 8), COPD (*n* = 8), colonoscopy-negative controls (CNC) (*n* = 8) and a panel of healthy controls (HC) (*n* = 20). Data were compared using one-way ANOVA adjusted for Tukey’s multiple comparisons test. Cohort 2 consisted of patients with NSCLC (*n* = 12), SCLC (*n* = 8) and healthy controls (HC) (*n* = 43). Data were compared using Kruskal-Wallis adjusted for Dunn’s multiple comparisons test. Cohort 3 comprised serum samples from patients diagnosed with IPF (*n* = 116) and healthy controls (HC) (*n* = 38). Groups were compared using unpaired, two-tailed Mann-Whitney test. Data are shown as Tukey box plots. Significance levels: ***: *p* < 0.001 and ****: *p* < 0.0001

DCN-CS were measured in serum samples from three independent cohorts including patients with lung cancer, IPF, COPD and healthy controls. The data are presented in Fig. 3. Results from cohort 1 show that DCN-CS was significantly elevated in serum from NSCLC (*p* < 0.0001) and IPF (*p* < 0.001) patients as compared to healthy controls. No significance was observed for COPD patients. The mean level of DCN-CS was also significantly higher in NSCLC patients compared to colonoscopy-negative controls, IPF and COPD patients. Data from cohort 2 confirmed the findings from cohort 1: DCN-CS was significantly elevated in NSCLC and SCLC patients as compared to healthy controls (*p* < 0.0001). Cohort 3 included patients with IPF and confirmed the results observed in cohort 1; IPF patients had a significantly higher level of DCN-CS (*p* < 0.0001) as compared to healthy controls.

**Fig. 4:**
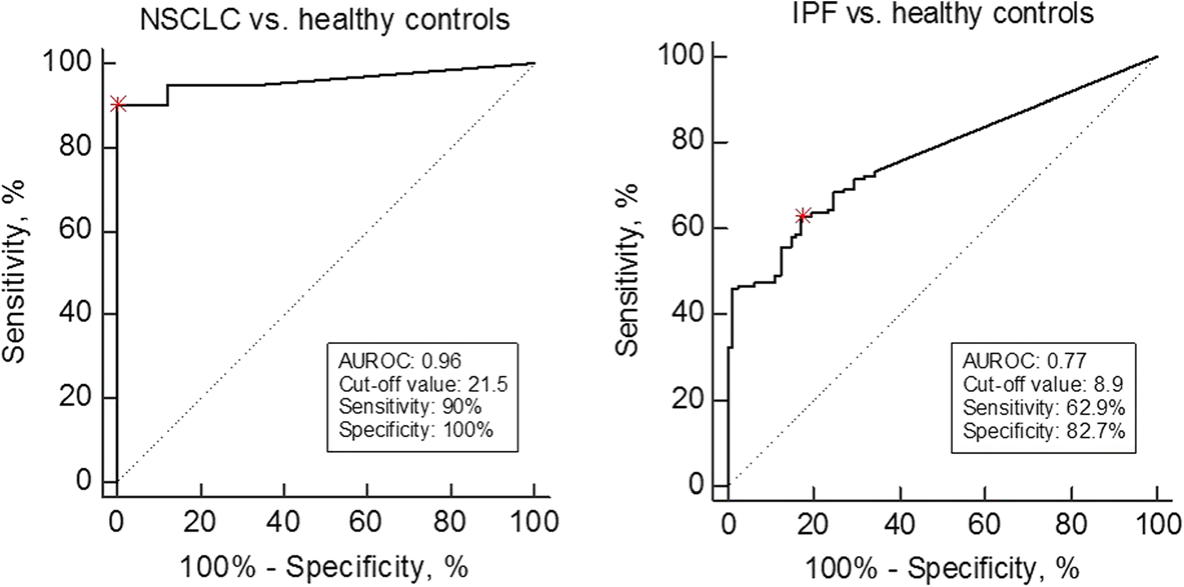
ROC curve analysis. Roc curve analysis was used to evaluate the ability of DCN-CS to discriminate between patients and healthy controls. The preliminary estimated cut-off values for the reported sensitivity/specificity are marked with a red asterix

The area under the receiver operating characteristics (AUROC) was used to evaluate the discriminative power of DCN-CS in relation to NSCLC and IPF. NSCLC patients, IPF patients and healthy controls from all cohorts were pooled and grouped into ‘NSCLC’, ‘IPF’ and ‘healthy controls’. As shown in Table 4, DCN-CS was able to discriminate between NSCLC patients and healthy controls with an AUROC of 0.96 (95%CI: 0.90–0.99), *p* < 0.0001) with a specificity of 100% and sensitivity of 90% for an estimated optimal cut-off value. Similarly, DCN-CS was able to identify IPF patients from healthy controls with an AUROC of 0.77 (95%CI: 0.71–0.83), *p* < 0.0001) with a specificity of 83% and sensitivity of 63% for an estimated optimal cut-off. The ROC curves are presented in Fig. 4.

**Table 4:** Discriminative performance of DCN-CS in NSCLC and IPF

	Cut-off value (ng/mL)	Sensitivity	Specificity	AUROC (95% CI)	*p*-value
NSCLC vs. healthy controls	21.5	90.0	100.0	0.96 (0.90–0.99)	<0.0001
IPF vs. healthy controls	8.9	62.9	82.7	0.77 (0.71–0.83)	<0.0001

These findings indicate that DCN-CS levels are able to separate patients with NSCLC and IPF from healthy controls with high diagnostic accuracy. Thus this specific fragment in serum has biomarker potential in fibrotic lung disorders such as lung cancer and IPF.

## Discussion

The present study describes the development and biological validation of a technically robust competitive ELISA assay quantifying a Cat-S degraded fragment of decorin in serum. The main findings of this study were: 1) the fragment was significantly elevated in lung cancer and IPF patients compared to healthy controls 2) the fragment was detectable in serum and 3) the assay was technically robust and specific towards a unique Cat-S degraded fragment of decorin, DCN-CS. To our knowledge this is the first biological validation of a specific decorin fragment in fibrotic lung disorders.

Decorin has been shown to play a protective role in cancer and fibrosis due to its ability to modulate various signal transduction pathways and sequester TGF-beta via direct binding [5]. This has led to the speculation that degradation of decorin may induce the development of cancer and fibrotic diseases by disrupting the binding to its binding partners. The lungs are an organ with a large amount of interstitial matrix and we have shown that patients with fibrotic lung disorders, such as cancer and IPF, have an increased level of degraded decorin. Cat-S is produced in both tumor cells and tumor-associated macrophages and has been associated with growth, angiogenesis and metastasis in different cancer types [26–28]. The interaction between these two proteins might give rise to a certain pathology where the interstitial matrix is involved and this interaction can be measured by the assay presented here.

The data suggest that Cat-S specific degradation of decorin has a relevant role in the pathology of lung cancer and IPF. We hypothesize that increased degradation of decorin triggers a fibrotic response both by inhibiting binding of TGF-beta to decorin which will result in the release of excessive amounts of TGF-beta but also by disrupting proper collagen fibril formation leading to loss of homeostasis in the interstitial matrix. Several studies have shown that disrupting the normal collagen turnover balance leads to fibrosis and cancer [1–3, 22]. Why the specific decorin fragment was not significantly elevated in patients with COPD is to be investigated further.

Based on the high elevated level of degraded decorin in patients compared to healthy controls, the present assay can provide a novel non-invasive clinical tool in lung cancer and IPF. The fact that the AUC was higher for lung cancer compared to IPF, suggests that this pathological event seems to be more associated with lung cancer than IPF and DCN-CS could serve as a potential diagnostic biomarker for lung cancer. In relation to IPF, the data suggests other clinical uses of this biomarker, such as prognosis and/or prediction. This needs to be further investigated in larger clinical studies. Evidence suggests that decorin fragments can function as pro-inflammatory signaling molecules, so-called damage-associated molecular patterns (DAMPs), capable of inducing an inflammatory response [5, 29]. High levels of degraded decorin might therefore indicate a severe inflammatory state. However further studies are needed to investigate whether the DCN-CS fragment functions as a DAMP.

The DCN-CS assay was shown to be technically robust, with low values of LLOD, intra- and inter variation and acceptable dilution recovery, interference and analyte stability at 4 °C. The fact that the assay did not detect the elongated peptide nor a non-sense peptide indicates that the monoclonal antibody is specific towards the cleavage site between amino acid 74 and 75 located in the first leucine-rich repeat of decorin. This was supported by data showing that DCN-CS was able to quantify high levels of the fragment after in vitro cleavage of decorin with Cat-S. Reactivity towards intact decorin was minimal further demonstrating that this assay does not measure total protein but a specific degraded fragment.

The target peptide fragment was originally identified by Zhen et al. [23] in human articular cartilage digested with ADAMTS-5. We have shown that this fragment is generated by Cat-S and not by Cat-L, MMP-2/9 or ADAMTS-5 in vitro using recombinant decorin. The fact that ADAMTS-5 degradation could not generate the target peptide fragment under our conditions, indicates that other proteases may have to cleave the protein before ADAMTS-5 can generate this fragment. Imai et al. [30] have examined the ability of different MMP’-s to cleave decorin and found that MMP-2, MMP-3 and MMP-7 were able to generate degradation fragments. None of these fragments correspond to our target fragment which supports our findings that the DCN-CS fragment is specifically generated by Cat-S and not MMP’s. This is important since different protein fragments may reflect different pathological events [31, 32], i.e. Cat-S degraded decorin might reflect one disease state whereas MMP-degraded decorin reflects other disease activity patterns. As the present assay enables quantification of a specific neo-epitope it might be superior to other commercial assays in which decorin is quantified but the precise epitope is not known. These quantification capabilities also increases the biomarker potential as it may reflect a direct pathological event, such as fibrosis.

The diagnostic validation of DCN-CS in the present study is limited by relatively small population sizes and cross-sectional designs and clinical information was limited. In addition, it was not easy to match cases and controls according to age, gender and tobacco consumption in this preliminary study which therefore could be confounding factors. However the fact that we could confirm the findings in independent cohorts, increases the validity. Larger longitudinal studies are needed to fully validate the potential of DCN-CS as a diagnostic and/or prognostic biomarker in fibrotic lung diseases.

## Conclusion

In conclusion, we have developed a technically robust competitive ELISA assay targeting a specific Cat-S degraded fragment of decorin (DCN-CS). The level of DCN-CS was significantly higher in patients with lung cancer and IPF compared to healthy controls, suggesting a pathological role of degraded decorin in these lung disorders.

## Abbreviations

ADAMTS: A disintegrin and metalloproteinase with thrombospondin motifs
AUROC: Area under the receiver operating characteristics
Cat-L: Cathepsin L
Cat-S: Cathepsin-S
COPD: Chronic obstructive pulmonary disease
DAMP: Damage-associated molecular pattern
DCN-CS: Decorin degraded by cathepsin-S
ECM: Extracellular matrix
EGFR: Epidermal growth factor receptor
ELISA: Enzyme-linked immunosorbent assay
GAG: Glycosaminoglycan
IPF: Idiopathic pulmonary fibrosis
IGF-IR: Insulin-like growth factor receptor
LLOD: Lower limit of detection
LLOQ: Lower limit of quantification
NSCLC: Non-small cell lung cancer
MMP: Matrix metalloproteinase
SLRP: Small leucine-rich proteoglycan
ULOD: Upper limit of detection
